# Supplementary material for: Nutrition and Immunity in Perinatal Hypoxic-Ischemic Injury
Source: Nutrients. 2022 Jul 1;14(13):2747. doi: 10.3390/nu14132747 (PMC9269416; doi:10.3390/nu14132747)
Supplement: Supplementary file 1 [file nutrients-14-02747-s001.zip › nutrients-1773236-supplementary.pdf]

Supplementary Material

Title: Nutrition and immunity in perinatal hypoxic-ischemic injury.

Authors: Gandecha Hema, Kaur Avineet, Sanghera Ranveer, Preece Joanna and Pillay Thillagavathie

Search engines used : Medline, EMBASE and PubMED
Time Span : 2012 -2022

**Concepts explored**	**Search strategy: key words/phrases**	**Total number of English language articles / abstracts identified**
Interplay between macronutrients and micronutrients and immunity	Nutrition AND immunity AND (micronutrient OR macronutrient)	363
Effect of hypoxia ischaemia on nutrition in relation to immunity	(Hypoxia OR ischemia OR ischaemia OR hypoxic) AND (Immunity OR Immune) AND (Trace Element OR Micronutrient OR Macronutrient)	84
Interplay between macronutrients and micronutrients and neonatal immunity	Immunity AND (Neonate OR Newborn OR Fetus OR Foetus) AND one of the following:<br>1. Vitamin A<br>2. Vitamin B (Thiamin / vitamin B1, riboflavin / vitamin B2, niacin / vitamin B3, pantothenic acid, vitamin B6, biotin / vitamin B7, folate / folic acid, vitamin B12).<br>3. Vitamin C<br>4. Vitamin D<br>5. Vitamin E<br>6. Folate<br>7. Zinc<br>8. Copper<br>9. Iron<br>10. Selenium<br>11. Glucose<br>12. Oligosaccharides<br>13. Amino Acids<br>14. Dietary nucleotides<br>15. Glycoproteins<br>16. Fatty acids OR Short chain fatty	Individual searches conducted to complete Table 1 (See individual references)

	acids OR long chain polyunsaturated fatty acids	
Therapeutic hypothermia and immunity	Newborn AND Therapeutic Hypothermia AND Immunity<br>Newborn AND Cooling AND Immunity<br>Neonate AND Therapeutic Hypothermia AND Immunity<br>Neonate AND Cooling AND Immunity<br>• After removal of duplicates	206
Hypoxia ischemia, nutrition and immunity in neonates	Hypoxia AND Ischaemia AND (Newborn OR Neonate OR infant)<br>AND<br>1. Brain injury<br>2. Brain<br>3. Mitochondria<br>4. Metabolism<br>5. Placenta<br>6. Glucose<br>7. Oxidative stress<br>8. Antioxidants<br>9. Free radicals<br>10. Oxidative damage<br>11. Ketones<br>12. Electrolytes<br>13. Iron<br>14. Hypoglycaemia<br>15. Hyperglycaemia<br>16. Brain energy	24
Interaction between nutrition and immunity and the microbiome in the newborn	Nutrition AND Immunity AND Microbiome AND (Neonate OR Newborn)	65
